# Metabolism and Biodegradation of β-Glucan *in vivo*

**DOI:** 10.3389/fvets.2022.889586

**Published:** 2022-06-03

**Authors:** Ziming Zheng, Wenqi Tang, Weipeng Lu, Xu Mu, Yuxuan Liu, Xianglin Pan, Kaiping Wang, Yu Zhang

**Affiliations:** ^1^Department of Pharmacy, Union Hospital, Tongji Medical College, Huazhong University of Science and Technology, Wuhan, China; ^2^Hubei Province Clinical Research Center for Precision Medicine for Critical Illness, Wuhan, China; ^3^Hubei Key Laboratory of Nature Medicinal Chemistry and Resource Evaluation, Tongji Medical College of Pharmacy, Huazhong University of Science and Technology, Wuhan, China; ^4^Department of Pharmacy, The Central Hospital of Wuhan, Tongji Medical College, Huazhong University of Science and Technology, Wuhan, China

**Keywords:** lentinan, degradation, metabolism, β-glucans, fluorescent labeling

## Abstract

The β-Glucans widely exist in plants and edible fungi, and their diverse bioactivities and good physicochemical properties have been widely reported. In addition, β-glucan intravenous injections (such as lentinan and schizophyllan) have been clinically used as immunomodulators and antitumor polysaccharides. However, the pharmacokinetic studies of β-glucans only stay on the level of plasma concentration and biodistribution *in vivo*, and little is known about their metabolism and degradation *in vivo*, which severely limits the further application of β-glucans in the field of medicine and biomaterials. The aim of this paper is to explore the metabolism and degradation process of lentinan (as a representative of β-glucans) *in vivo* by labeling it with water-soluble fluorescein 5-([4, 6-Dichlorotriazin-2-yl]amino)fluorescein (DTAF). Fluorescently labeled lentinan (FLNT) was intravenously administered to rats at a single dose of 8 mg/kg. The degradation of LNT in blood, liver, kidney, and urine was evaluated by the gel permeation chromatography. Our results showed that although LNT could be degraded in blood, liver, kidney, and urine, there were still some prototypes until excreted in urine due to the incomplete degradation of LNT in each step. To the best of our knowledge, this is the first report to comprehensively study LNT metabolic degradation in rats. These results provide an important reference for further exploration and application of LNT and other β-glucans.

##  Introduction

Lentinan (LNT), which is a purified β-1,3-glucan with β-1,6-branches isolated from *Lentinus edodes*. LNT, which is known for its immune activity like other β-glucans from medicinal mushrooms (1–3), has been reported as an intravenous antitumor polysaccharide *via* enhancement of the host immune system (4). The clinical efficacy of LNT, such as its effect on long-term survival and improvement of life quality, has been confirmed in cancer patients (5, 6). LNT, a natural extract product with a unique advantage of multiple pharmacological activities and less toxicities, has attracted more and more concerns. However, the main studies focused on structure characterization, clinical effects, immune mechanisms, and new bioactivities (7–11), the pharmacokinetics *in vivo* were rarely reported. Furthermore, the pharmacokinetics information in the instruction of LNT injection, which has been approved for listing and used in clinical, was described incompletely. It has been reported that there was a dose–response relationship between β-glucans (such as LNT) and their pharmacological activities (12, 13). In another study, the treatment for advanced cancers would be better administered at a higher dose of lentinan than the conventional dose (14). On the other hand, the side effect of LNT was linked with the dose and rapid administration (12, 14). All of these reports were closely related to the pharmacokinetics of LNT. Several lines of statement emphasize that the blameless pharmacokinetics information is important and urgent for the clinical application of LNT.

In general, the pharmacokinetic studies of β-glucans have mainly focused on the blood concentration-time profiles and tissue distribution (15–17). Whether β-glucans are metabolized or degraded is rarely reported, and the related opinions are controversial (18, 19). Previously, β-glucans were often thought not to be metabolized or degraded in the body due to the lack of β-glucanase (20). However, subsequent studies have demonstrated that β-glucans could be metabolized in the liver and abdominal cavity (18, 21).

Our previous work showed that LNT extracted from *Lentinus edodes* was a typical triple-helical β-glucan and exerted good antitumor activity (9, 22). To supplement the pharmacokinetic information, LNT was labeled with 99 m-technetium (^99m^Tc) and intravenously injected into animals to evaluate its plasma concentration, biodistribution, and excretion. The dynamic emission tomography imaging of ^99m^Tc-labeled LNT showed that LNT in the blood rapidly accumulated in the liver and was then mainly excreted in the urine through the kidneys (15). However, whether LNT was metabolized or degraded *in vivo* has not been studied. The γ signal represented the LNT prototype or its degraded fragment, which attracted our great interest. The concentration and molecular weight (Mw) of LNT determined its viscosity in the blood, thereby affecting its side effects (12). Therefore, understanding whether LNT is degraded in the blood can provide a reference for its clinical application. It has been reported that the Mw of polysaccharides was closely related to its excretion in the kidneys (23). It can be speculated that if LNT is degraded in the kidney, it will be beneficial to its excretion. In order to fully understand the whole process of LNT metabolic degradation *in vivo*, in this paper, LNT was labeled with 5-([4,6-Dichlorotriazin-2-yl]amino)fluorescein (DTAF) to determine its degradation in blood, liver, kidney, and urine by high-performance gel permeation chromatography (HPGPC). The comprehensive pharmacokinetic study *in vivo* is important for the development and application of LNT and other β-glucans.

## Materials and Methods

### Materials

The dried fruit body of *Lentinus edodes* was purchased from Fangxian, Hubei Province, China. It was identified as *Lentinus edodes* by Professor Jiachun Chen from the Department of Traditional Chinese Medicine, Tongji Medical College at Huazhong University of Science and Technology. DTAF was obtained from ATT Bioquest (Sunnyvale, USA). All chemicals used in this study were of analytical grade.

### Animals

Healthy female Sprague–Dawley (SD) rats weighing 200 ± 20 g were obtained from the Experimental Animal Center of Tongji Medical College, China (Reg. no. SCXK [Hubei] 2016-0009). The rats were housed with free access to food and water, and maintained in an air-conditioned room (25 ± 2°C, relative humidity 50 ± 20%, 12 h light/dark cycle). Animal welfare and experimental procedures were approved (ID: SYXK (Hubei) 2021-0057) by the Institutional Animal Care and Use Committee of Huazhong University of Science and Technology (Wuhan, China).

### Extraction and Characterization of LNT

Lentinan was extracted and purified according to our previous studies (24). Briefly, the dried fruit bodies of *Lentinus edodes* were first treated with 95% alcohol and boiling water. The fruit body residues were soaked in 2% NaOH solutions to obtain the crude polysaccharides. The crude polysaccharides were decolorized and purified sequentially by ultrafiltration and Sephadex G-100 chromatography to obtain LNT. The Mw of LNT was determined by HPGPC as described in our previous report (24). Briefly, a TSK-GEL G-4000PW_XL_ column was maintained at 35°C, and the mobile phase was a 0.1 M NaNO_3_ solution at a flow rate of 1.0 ml/min. T-series Dextrans (1–2000 kDa) were used to determine the standard curve, and the Mw of LNT was calculated with the Agilent HPGPC software (Agilent, USA) according to the calibration curve equation. The ultraviolet (UV) and Fourier transform infrared (FT-IR) spectra analyses were performed according to our previous studies (11, 25). Brie?y, the UV spectrum of the LNT solution (1 mg/ml) was recorded with a UV spectrophotometer in the region of 200–800 nm for detecting proteins and nucleic acids. The infrared spectrum was recorded with KBr pellets on a Bruker-VERTEX 70 FT-IR spectrophotometer in the 4000–400 cm^−1^ region.

### Synthesis of Fluorescently Labeled Lentinan (FLNT)

Lentinan (40 mg) was dissolved in carbonate buffer (20 ml, pH = 9.5). The DTAF (3 mg) was added to the solution and incubated at room temperature and 4°C with stirring for 24 h, respectively. The mixtures were dialyzed against distilled water to remove salt and free DTAF. For further purification, the samples were loaded onto a Sephadex G-50 chromatography column and eluted with distilled water.

### Toxicity of FLNT *in vivo*

A single dose (8 mg/kg) of FLNT or LNT was administered intravenously to rats through the tail vein. After administration of 24 h, the rats were anesthetized by injection of 3% sodium pentobarbital (50 mg/kg), and then sacrificed and dissected. Heart, liver, spleen, lung, and kidney embedded in paraffin were cut into 4 μm slices for staining with hematoxylin and eosin (H&E). Histological lesions of these organs were compared in H&E-stained sections to judge the toxicity of FLNT and LNT.

### Stability of FLNT *in vitro*

Fluorescently labeled lentinan (100 μg/ml) was incubated in phosphate-buffered saline (PBS) at 37°C for 24 h, and then double volume saturation ammonium sulfate [(NH_4_)_2_SO_4_] solution was added. After being mixed and centrifuged at 12,000 rpm for 10 min, 20 μl of the supernatant was taken for HPGPC analysis according to our previous studies (25). Briefly, WAT011545 (Waters, Japan) column was maintained at 35°C, and the mobile phase was 0.1 M NaNO_3_ solution at a ?ow rate of 1.0 ml/min. The detection signal was recorded by a fluorescence detector (RF-20A).

### Degradation of FLNT in Blood

A single dose (8 mg/kg) of FLNT was administered intravenously to rats through the tail vein. After administration, the rats were anesthetized by injection of 3% sodium pentobarbital, and blood samples (approximately 500 μl each) were obtained periodically (5, 15, 30, and 60 min) through the orbital canthus. After standing for 2 h, the samples were centrifuged at 4°C for 10 min to collect the supernatant. For *in vitro* experiment, FLNT solution was mixed with a 9-fold volume blank blood and incubated at 37°C. The samples were obtained periodically at 5 min, 0.5, 2, and 24 h. For above *in vivo* and *in vitro* samples, two volume of saturated (NH_4_)_2_SO_4_ solution was added and centrifuged (12,000 rpm at 4°C for 10 min) for protein removal. The Mw of FLNT collected from blood was determined by HPGPC as 2.5 stated.

### Degradation of FLNT in Liver and Kidney

A single dose of FLNT was applied intravenously to rats. After administration, the rats were anesthetized at a predefined time (0.5, 2, and 8 h post-dose). Liver and kidneys were promptly excised, washed, weighed, and homogenized with 4-fold volume PBS. Then the samples were centrifuged (7,500 rpm) at 4°C for 15 min to collect the supernatant. For *in vitro* experiment, FLNT solution was mixed with a 9-fold volume blank tissue homogenates and incubated at 37°C. The samples were obtained periodically at 0.5, 2, and 24 h. The treatment and detection of samples were carried out in accordance with Section 2.6.

### Degradation of FLNT in Urine

A single dose of FLNT was applied intravenously to rats. After administration, rats were held in stainless-steel metabolic cages for urine collection at 0–4 and 4–8 h after dosing. For *in vitro* experiment, FLNT solution was mixed with 9-fold volume blank urine and incubated at 37°C. The samples were obtained periodically at 5 min, 0.5, 2, 8, and 24 h. The treatment and detection of samples were carried out in accordance with Section 2.6.

## Results and Discussion

### Characterizations of LNT

**Figure 1:**
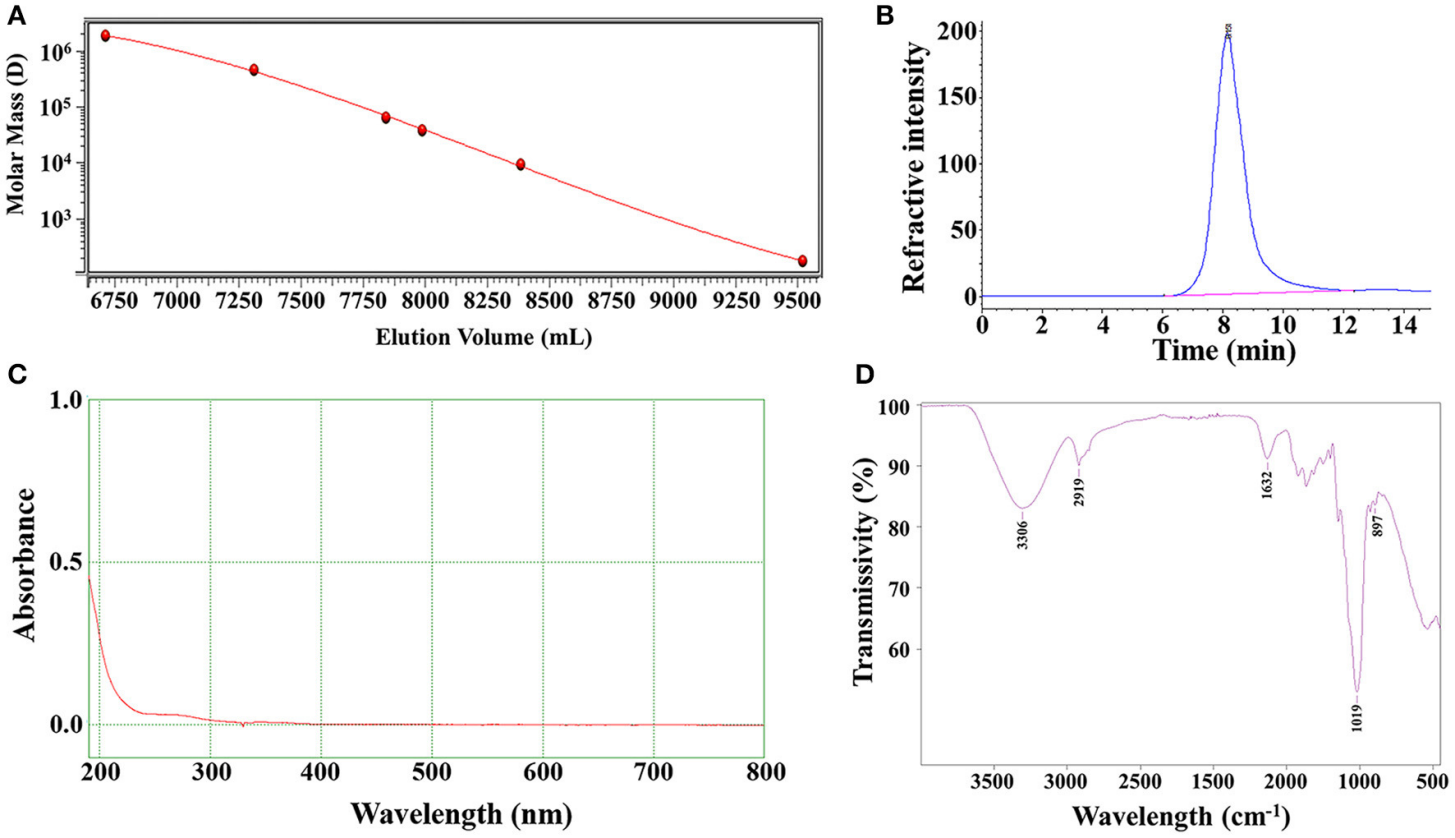
Characterizations of lentinan (LNT). **(A)** Molecular weight standard curve; **(B)** The chromatographic peak of LNT detected by the refractive index detector; **(C)** The ultraviolet (UV) spectra of LNT; **(D)** The Fourier transform infrared (FT-IR) spectra of LNT.

In order to ensure the quality of LNT was extracted from different batches, the Mw and uniformity of each batch of LNT were tested. The weight-average Mw of the LNT from HPGPC analysis was calculated as 432.6 kDa and showed good homogeneity (Figures 1A,B). No UV/Vis absorption peaks were observed at 260 and 280 nm (Figure 1C), indicating that LNT contained no proteins or nucleic acids. As shown in Figure 1D, the bands of FT-IR spectrum around 3,306 and 2,919 cm^−1^ were due to the hydroxyl stretching vibration and C–H stretching vibration, respectively. Bands around 1,632 cm^−1^ represented the H–O–H bending vibration (26). All of these peaks were characteristic absorption peaks of polysaccharides. The absorption peaks of β-glycosidic bonds and α-glycosidic bonds are around 890 and 840 cm^−1^, respectively. Bands around 897 cm^−1^ indicated that LNT mainly contained β-glycosidic bonds. These results were in line with the quality standard of LNT as β-glucan.

### Synthesis of FLNT

**Figure 2:**
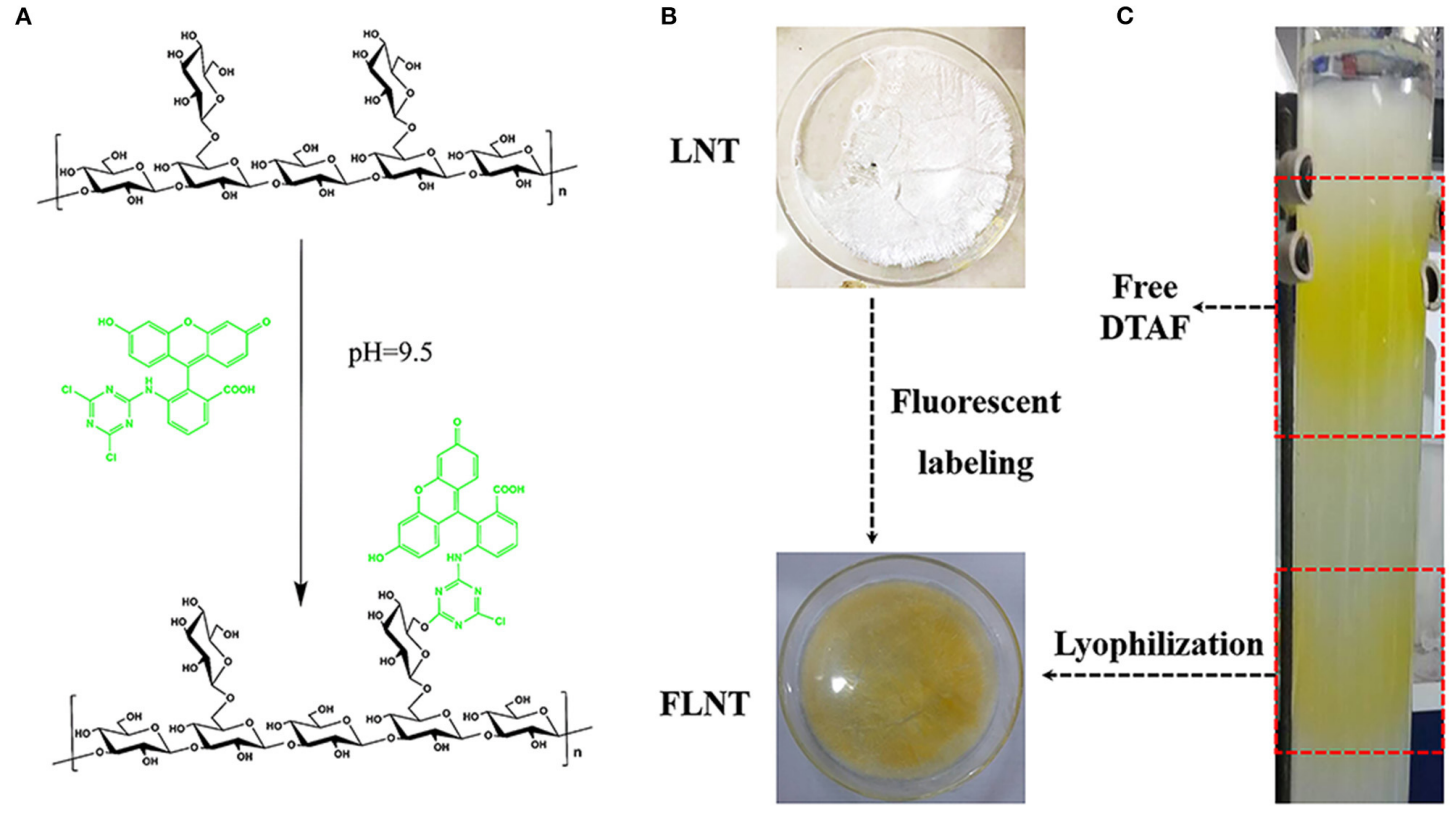
Synthesis of fluorescently labeled lentinan (FLNT). **(A)** Scheme of LNT and 5-([4,6-Dichlorotriazin-2-yl]amino)fluorescein (DTAF) reaction. **(B)** The picture of LNT and FLNT. **(C)** The reaction solution was purified by the Sephadex G-50 column.

The nucleophilic reaction of LNT hydroxyl groups with DTAF can only occur in an alkaline environment with pH ≥ 9. However, DTAF will degrade if the pH of the reaction system exceeds the p*K*a of DTAF at 10.82 (27). So, pH = 9.5 carbonate buffer consisting of sodium carbonate and sodium bicarbonate was chosen as the reaction solution in this work (Figure 2A). Free DTAF was removed by dialysis against deionized water followed by size exclusion chromatography on Sephadex G-50 (Figures 2B,C). According to the separation principle of the molecular sieve effect, the lower layer was represented FLNT with larger Mw, and the upper layer was represented free DTAF with smaller Mw. The triple helical structure of LNT is easily transformed into single flexible chains in organic solvents, such as dimethyl sulfoxide (DMSO) (28). Therefore, traditional FITC-derived polysaccharides may not be suitable for LNT labeling. The DTAF is a water-soluble fluorescein, and the reaction system with LNT does not require organic solvents, thereby reducing the impact on its triple helical structure.

### Toxicity of FLNT *in vivo*

**Figure 3:**
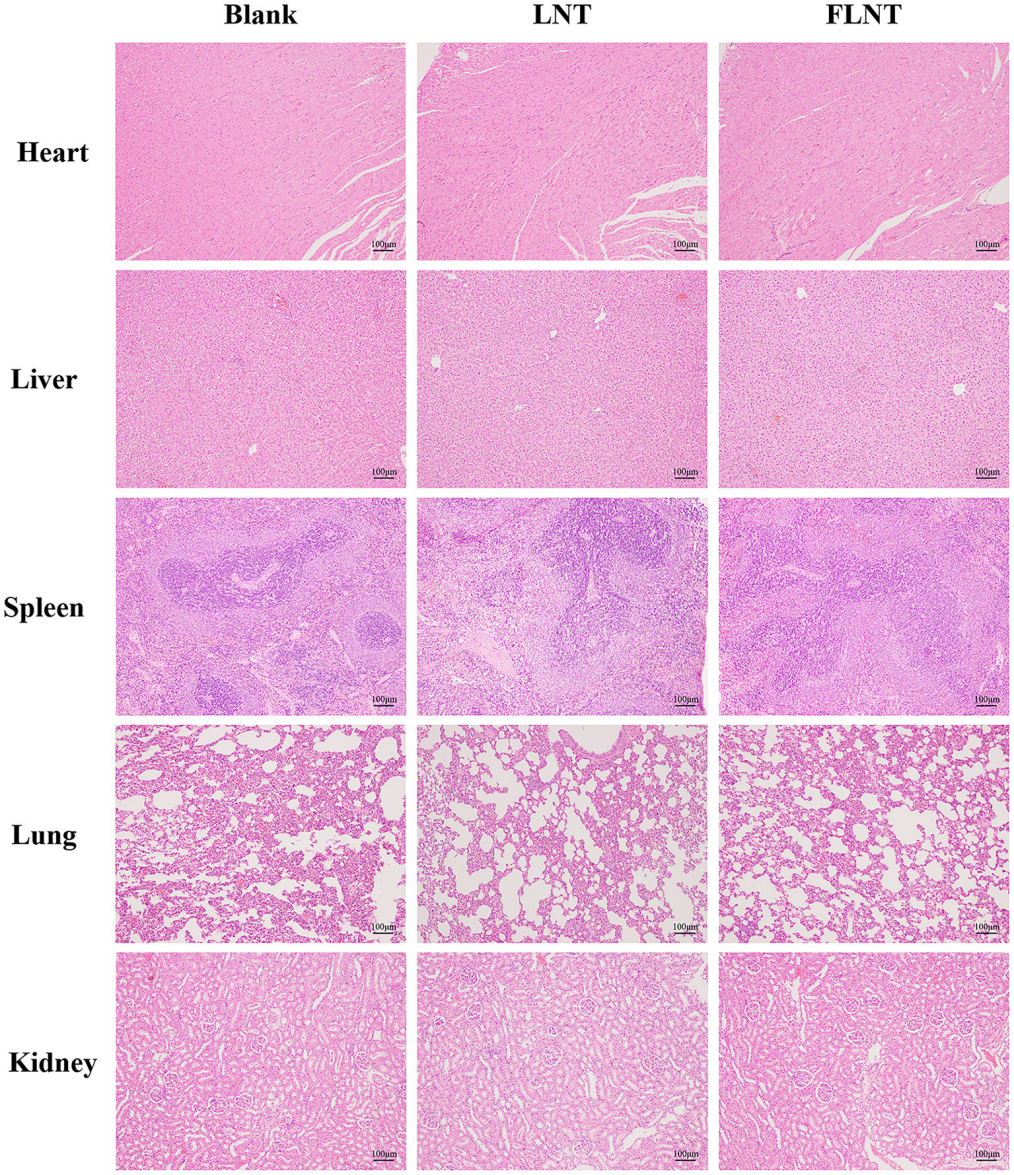
Hematoxylin and eosin staining of organs (bar = 100 μm).

In this study, the survival rate of mice was 100% after a single intravenous dose of 8 mg/kg. In our previous study, we found that no mice were died after the continuous intravenous dose of 5 mg/kg for 21 days (11). These data confirmed that LNT was safe, consistent with reported safety of other β-glucans (29). However, the safety of FLNT needs further confirmation. As shown in Figure 3, consistent with the blank control group, the heart, liver, spleen, lung, and kidney of the rats in LNT or FLNT groups were structurally complete; the cells were neatly arranged and normally distributed. No obvious toxicological damage was found in main organs after single-dose administration of LNT and FLNT.

### Stability of FLNT *in vitro*

**Figure 4:**
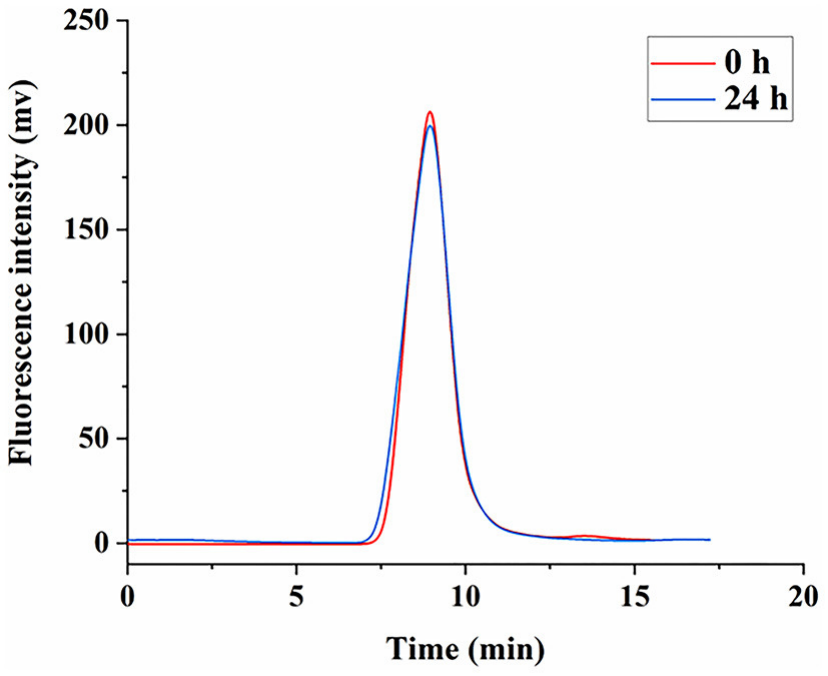
Stability of FLNT incubation in phosphate-buffered saline (PBS) solution at 37°C.

After the fluorescent labeling of LNT, the HPGPC fluorescence chromatogram was used to judge the degradation of FLNT. In order to ensure that the temperature and the protein removal conditions have no effect on the degradation of LNT, the stability of FLNT was investigated. As shown in Figure 4, there was no degradation when FLNT was incubated with PBS at 37°C for 24 h, followed by being treated with 2-fold volume saturation (NH_4_)_2_SO_4_ solution, indicating that the stability of FLNT was good.

### Metabolic Degradation in Blood

**Figure 5:**
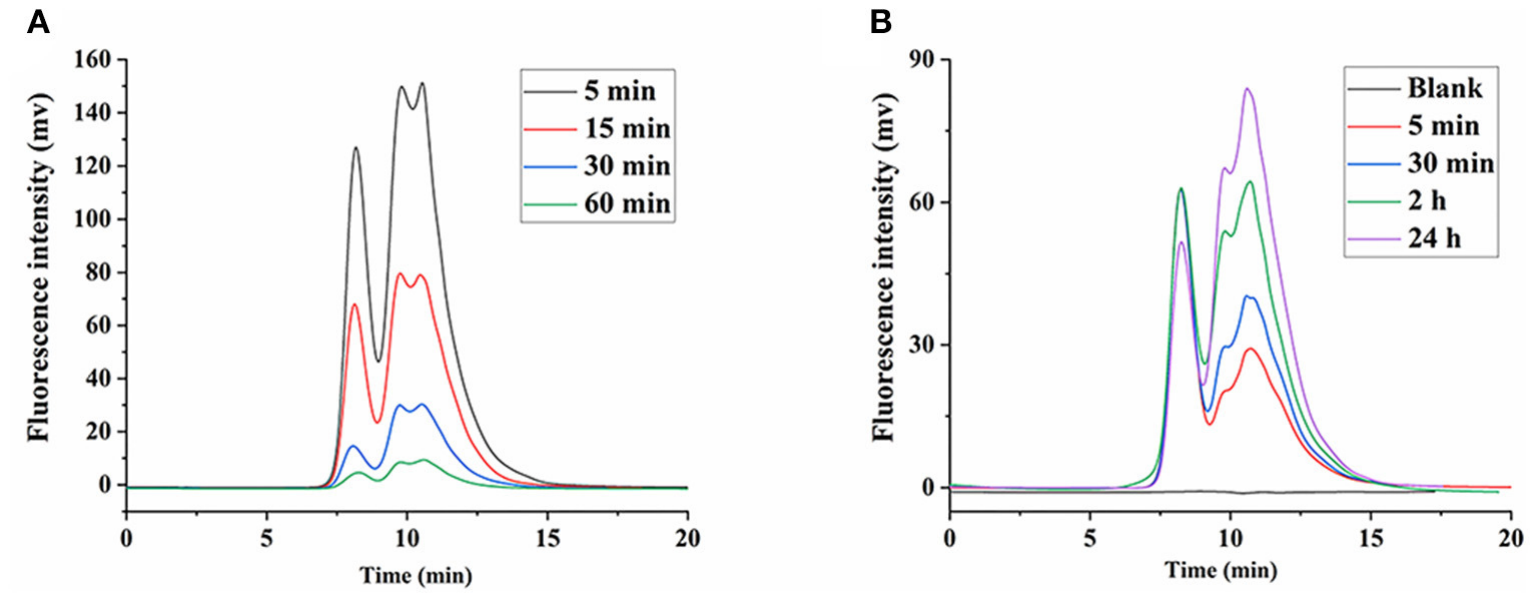
Degradation of FLNT in blood. **(A)** Degradation of FLNT at different time points in blood *in vivo*. **(B)** Degradation of FLNT at different time points in blood *in vitro*.

Initially, considering the lack of glucanase, it was thought that β-glucans were not degraded in the body and were excreted from the body as their prototypes. However, some studies found that β-glucans were degraded in the body (30, 31). Although LNT was quickly concentrated in the liver after intravenous administration (15), the Mw of LNT affected its viscosity in the blood, which was closely related to its side effects (12). As shown in Figure 5A, with the increase of time, the peak intensity of FLNT significantly decreased, indicating that the content of FLNT in the blood gradually decreased. Interestingly, there was a significant degradation even at 5 min, and its degradation trend was not significantly different compared with 1 h. This could be attributed to the fact that the blood samples stood for 2 h before centrifugation. Therefore, even if the blood was obtained at 5 min, FLNT was left in the blood for sufficient time to be degraded.

In order to eliminate the error in degradation time caused by standing, the *in vitro* degradation of FLNT in rat plasma was studied. As shown in Figure 5B, as the incubation time increased, the intensity of the degradation peak significantly increased. There were still many prototypes of FLNT at 24 h, indicating that FLNT degraded slowly in the plasma. It can be found that the FLNT degradation peak characteristics in plasma *in vitro* were different from those *in vivo*, indicating that in addition to enzymes of plasma involved in the degradation of LNT, blood cells might also participate in the metabolic degradation of LNT. It has been reported that immune cells (such as macrophages, neutrophils, and NK cells) in the blood could recognize the β-glucans on the surface of fungi to achieve the purpose of degrading and removing antigens (32, 33). These immune cells may participate in the degradation of LNT, which needs to be further studied. Nevertheless, there was a large number of prototype FLNTs that were not degraded in the plasma both *in vivo* and *in vitro*. Considering that FLNT quickly concentrated in the liver, it was speculated that a large number of FLNTs was taken up by the liver before being degraded in the blood, and there was a large number of prototype FLNTs in the liver.

### Metabolic Degradation in Liver

**Figure 6:**
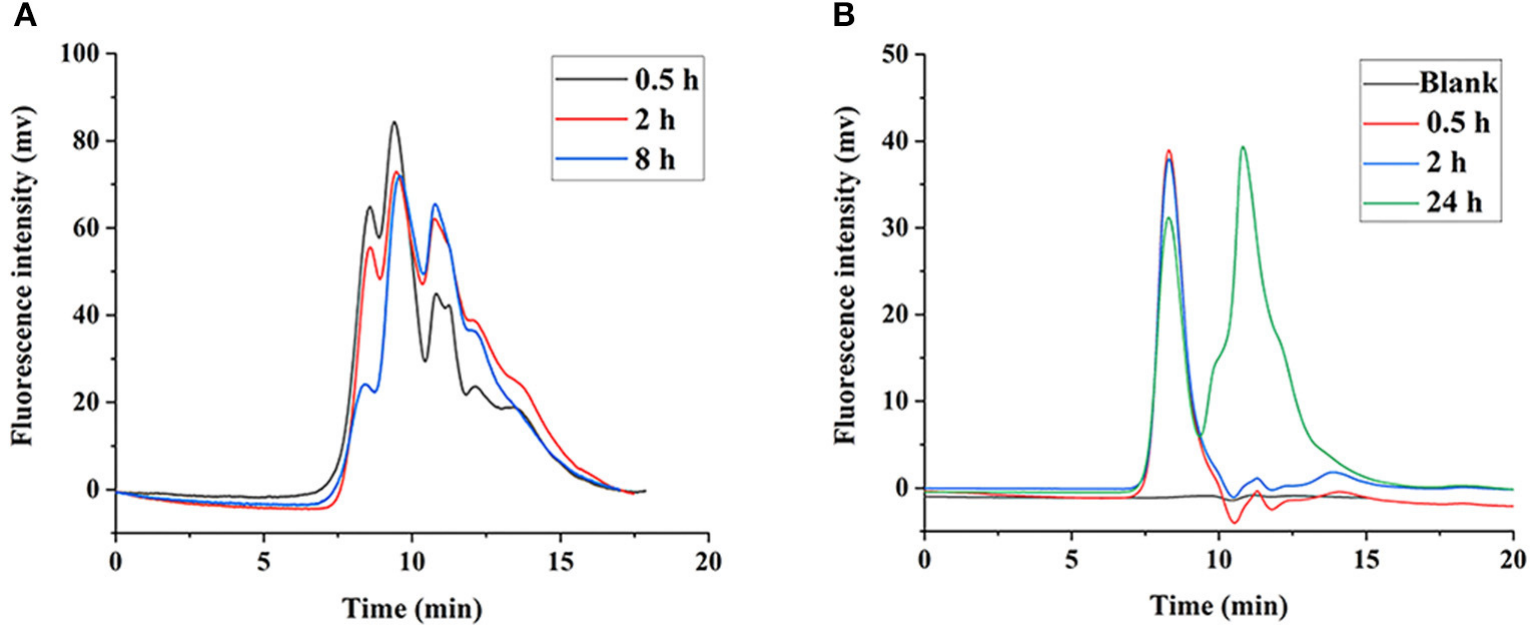
Degradation of FLNT in liver. **(A)** Degradation of FLNT at different time points in liver *in vivo*. **(B)** Degradation of FLNT at different time points in liver homogenate *in vitro*.

It was shown that the LNT mainly accumulated in the liver in our previous work (15), thus we speculate that the liver is the main site for LNT metabolism and degradation. The FLNT was recovered from liver at 0.5, 2, and 8 h after intravenous administration. The results showed that the FLNT was significantly degraded in the liver by HPGPC analysis (Figure 6A). However, there was still a large amount of prototype remained at 8 h, indicating that the FLNT was degraded slowly in the liver. To exclude the effect of blood on the liver metabolism of FLNT, perfused liver homogenates were used to incubate with FLNT at 37°C to determine the metabolic degradation of FLNT in the liver. The data indicated that the FLNT was significantly degraded in liver homogenate at 24 h (Figure 6B). These results suggested that the LNT could be degraded in the liver. The FLNT exhibited significant liver degradation *in vivo* at 0.5 h, while there was no significant degradation in perfused liver homogenate within 2 h. There are two reasons for this difference. On the one hand, the LNT has been degraded in the blood to a certain extent, and there were a certain proportion of degraded fragments when FLNT accumulated in the liver. On the other hand, the degradation of LNT in the liver may be related to the Kupffer cells (21), while liver homogenization led to a large number of cell death.

### Metabolic Degradation in Kidney and Urine

**Figure 7:**
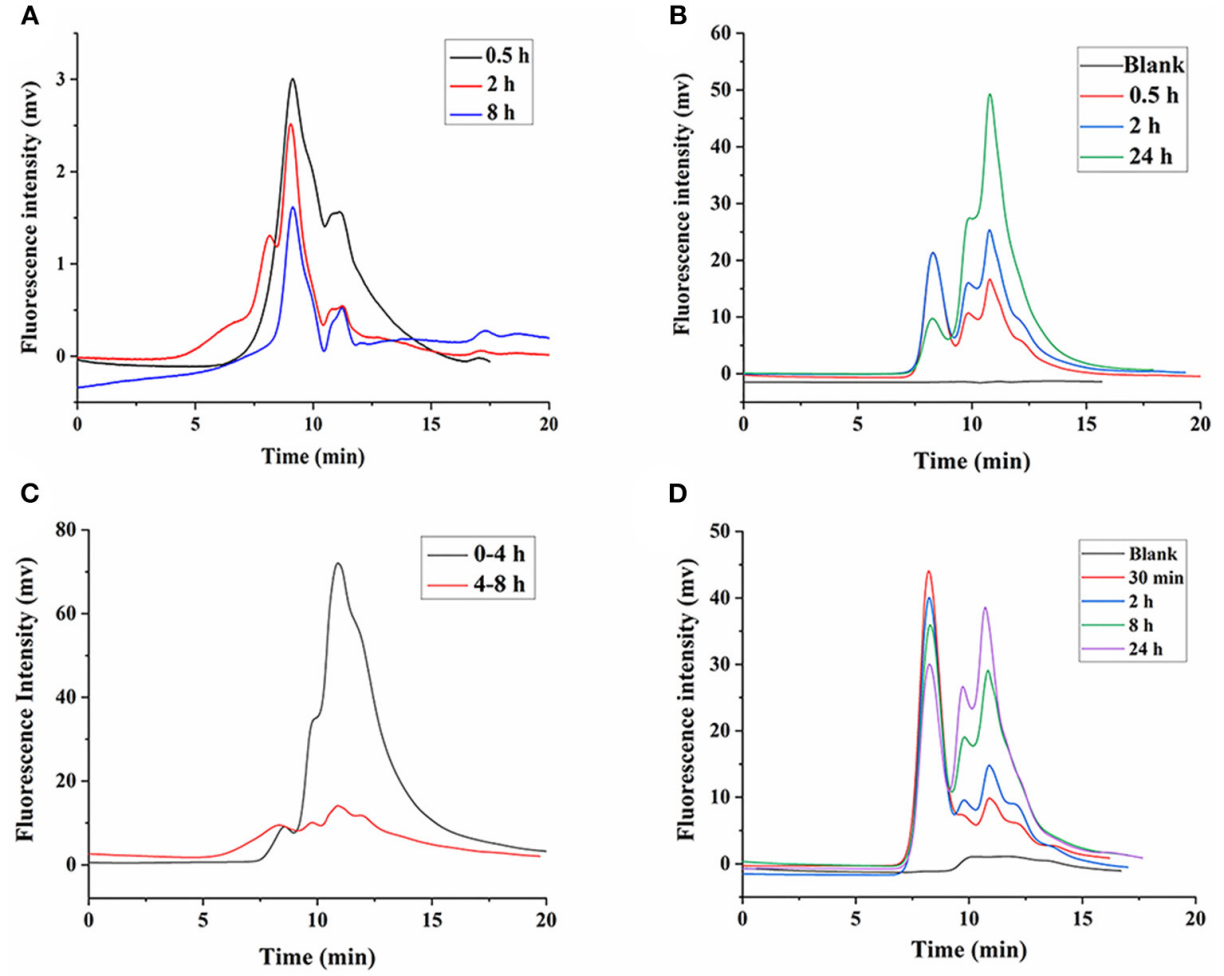
Degradation of FLNT in kidney and urine. **(A)** Degradation of FLNT at different time points in kidney *in vivo*. **(B)** Degradation of FLNT at different time points in kidney homogenate *in vitro*. **(C)** Degradation of FLNT at different time points in urine *in vivo*. **(D)** Degradation of FLNT at different time points in urine *in vitro*.

Our previous study showed that LNT is mainly excreted in urine through the kidneys in rats (15). Earlier reports indicated that renal excretion was the major pathway for polysaccharides with low Mw, whereas polysaccharides with high Mw were excreted from the liver, which was determined by the pore size of the glomerular capillary wall (34, 35). Intriguingly, urinary excretion played a major role in the elimination of high-Mw LNT in rats (15). Therefore, it could be inferred that LNT was metabolized or degraded into small molecular fragments *in vivo*, which was more conducive to excretion from the kidneys. As shown in Figure 7A, FLNT mainly existed as degraded fragments in the kidney, with few prototypes. In order to further explore whether LNT was degraded in the kidney, the FLNT was incubated with kidney homogenate. The results showed that FLNT could also be degraded in the kidney, and it was significantly degraded at 0.5 h (Figure 7B), indicating that its degradation rate was higher than that in the liver. However, there was still a certain amount of prototypes even at 24 h.

As shown in Figure 7C, FLNT was rapidly excreted to urine as degraded fragments within 4 h, but there were still a few of prototypes. During 4–8 h, the amount of FLNT excreted in urine decreased, and the proportion of FLNT degraded fragments significantly decreased. These results suggested that there were a large amount of FLNT-degraded fragments in the body during the first 4 h (possibly earlier), and these small molecular fragments were more likely to be excreted in the urine, which confirmed our previous research on LNT excretion rate (15). The samples after 8 h were not collected because the FLNT content was too low to be detected. The results of *in vitro* incubation of FLNT in urine showed that LNT also be degraded in urine (Figure 7D). Lysozyme has been reported to degrade polysaccharides in the kidneys and urine (36). In addition, it has been reported that β-glucan could enhance the lysozyme levels in the body (37). Therefore, lysozyme may be one of the reasons for the degradation of LNT in kidneys and urine.

## Conclusion

Based on the comprehensive analysis of the degradation characteristics of blood, liver, kidney, and urine *in vivo* and *in vitro*, the entire metabolic degradation process of LNT in rats after intravenous administration can be summarized. LNT will be partially degraded in the blood, but most of the prototypes will be concentrated in the liver. LNT in the liver is slowly degraded into fragments and then transferred to the kidneys. The remaining LNT in blood will also be distributed to the kidneys along with the circulatory system, and continue to be degraded in the kidneys. Finally, LNT will be further degraded by urine in the bladder and then excreted from the rats. When LNT is finally excreted in urine, there are still some prototypes due to the incomplete degradation of LNT in each step. To the best of our knowledge, this is the first report to comprehensively study LNT metabolic degradation in rats. This work provides a new option for investigating the metabolic degradation of β-glucans *in vivo*.

This study still has its limitations. In order to reduce the influence of the reaction system on the structure of β-glucans, water-soluble fluorescein was used to label LNT in this study. Therefore, this study is only valid for soluble β-glucans. There are still abundant insoluble β-glucans in nature, and new methods need to be explored to study their metabolism and degradation. In addition, other soluble β-glucans also need to be studied to verify the common features of soluble β-glucans metabolism and degradation.

## Data Availability Statement

The original contributions presented in the study are included in the article/supplementary material, further inquiries can be directed to the corresponding authors.

## Ethics Statement

The animal study was reviewed and approved by Institutional Animal Care and Use Committee of Huazhong University of Science and Technology (Wuhan, China).

## Author Contributions

YZ and KW initiated the research. WL and XM performed the data analysis. WT and YL prepared the lentinan. ZZ performed the fluorescent labeling work and animal experiments. ZZ and YZ prepared the original draft. XP and KW reviewed and edited this manuscript. All authors contributed to the article and approved the submitted version.

## Funding

This work was supported by the National Natural Science Foundation of China (Grants No. 82104282, 81974509, and 82074111).

## Conflict of Interest

The authors declare that the research was conducted in the absence of any commercial or financial relationships that could be construed as a potential conflict of interest.

## Publisher's Note

All claims expressed in this article are solely those of the authors and do not necessarily represent those of their affiliated organizations, or those of the publisher, the editors and the reviewers. Any product that may be evaluated in this article, or claim that may be made by its manufacturer, is not guaranteed or endorsed by the publisher.
